# A Manual of Surgical Treatment

**Published:** 1900-03

**Authors:** 


A Manual of Surgical Treatment. By W. Watson Cheyne, M. B , F. R. C. S., F. R S., Professor of Surgery in King's College, London ; Surgeon to King's College Hospital, etc , and F. F. Burghard, M. D. and M. S. (Lond ), F. R. C. S , Teacher of Practical Surgery in King's College, London ; Surgeon to King's College Hospital, etc. Volume II. 382 pages, with 141 illustrations. Philadelphia and New Yerk : Lea Brothers & Co. 1900.

If one has ever had the extreme good fortune to eat a Chinese dinner—a real Chinese dinner, not a luncheon, then one has some sort of an idea of some of the sensations of enjoyment and surprise experienced in receiving the second volume of this manual of surgical treatment, by Cheyne and Burghard. Chinese dinners are famous for their surprises, and this work is full of anticipation which is outstripped by realisation, giving it a sort of a friendly feeling. There is an air of confidence in the method of handling subjects which, to say the least, is grateful and comfortable in these days of high falutive rhetorical strain. In the second volume the authors have kept close to their original plan of simplicity, intelligibility and common sense, and there is in no place in the book any effort to display excessive and dispiriting surgical knowledge.

The student in going over this work instinctively feels that it is the product of men who know what they are writing; that it is all practical, that the procedures are proper and beyond the experimental stage and that there need be not the slightest hesitation in following the advice given in any case under consideration. It is all so plain, so exquisitely descriptive, and, again, one is told not only how to do things, but why and when.

Surely it is the exemplification of modern text-book writing, and better medicine, more intelligible treatment will be taught and infinitely better results will be achieved, when there is published a series of small volumes, such as these are in make-up, dealing with the practice of medicine in much the same way as these authors deal with surgery. Lydston has done genito-urinary work in one volume in this style; a style that impresses upon the casual reader the fact that there is much more in genito-urinary work than writing prescriptions to combat Neisser's low-bred and very common organism, or the passing of a sound.

In the recent volume of Cheyne and Burghard's work there is taken up the surgical affection of the tissues, including the skin and subcutaneous tissues, the nails, the lymphatic vessels and glands, the fasciæ bursæ, muscles, tendons and tendon sheaths, nerves, veins arteries and deformities.

In the preface the authors speak of some hypercritical reviewer's complaint that they had dealt with treatment independently of pathology and symptoms. The fault is not with the authors, but with the reviewer who went out of his way to air himself, for is not this a manual of surgical treatment, and did not the authors say in the preface in the first volume: "For the practitioner, on the other hand, who is already acquainted with these points (pathology, symptomatology and diagnosis) the great essential is full and detailed information as to the best methods of treatment?"

Again, as in the first volume, in the book just issued the text is made clearer, and the treatment made more intelligible, by the use of excellent illustrations. There is nothing left to the imagination; it is all taught. Not satisfied with saying that sutures should be placed thus and so, cuts are used to illustrate the manner and method of the placing. There is so little to criticise in the second volume that it is not worth while to say anything regarding the shortcomings of the book; they are so infinitesmal. The volume bears out all the promise of a great work given by the first, and adds interest and whets the appetite for those which are to follow. Provided the pace set by volumes I. and II. is kept up, the finished work will constitute one of the most valuable contributions to surgical literature published in years—valuable because it is good, plain, common-sense, every-day treatment, without any frills. N. W. W.

